# Predicting cervical insufficiency in twin pregnancies using ultrasound cervical measurements and elastography

**DOI:** 10.7150/ijms.99444

**Published:** 2024-11-11

**Authors:** Yi Huang, Qi Li, Weishe Zhang, Kuifang Shen, Jiahao Zhu, Hongtao Zeng, Xiuqing Lv, Jingrui Huang

**Affiliations:** 1 Department of Obstetrics, Xiangya Hospital Central South University, Changsha, China.; 2 Reproductive Medicine Center, Xiangya Hospital Central South University, Changsha, China.; 3 Hunan Engineering Research Center of Early Life Development and Disease Prevention, Changsha, China.

**Keywords:** uterine cervical incompetence, twin pregnancy, risk factors, elasticity imaging techniques

## Abstract

**Background:** To evaluate the predictive effect of transvaginal ultrasound measurement of cervical length and cervical elasticity examination on cervical insufficiency in twin pregnancies.

**Methods:** Data from twin pregnant women in our hospital were collected retrospectively, including relevant vaginal ultrasound parameters (e.g., cervical length, cervical elasticity score, and the strain value of each part of the cervix). We assessed the risk factors using receiver operating characteristic curve analysis to evaluate the predictive effect of each factor on the occurrence of cervical insufficiency.

**Results:** A total of 284 pregnant women with twin pregnancies, including 142 with cervical insufficiency and 142 without cervical insufficiency, were included. Significant differences between the two groups were observed in the use of assisted reproductive technology, age, history of second-trimester miscarriage, etc. The cervical length of pregnant women with cervical insufficiency was significantly shorter at 22-24 weeks of gestation. Cervical length had the largest area under the receiver operating characteristic curve for predicting cervical insufficiency at that time. The area under the curve of cervical insufficiency predicted by the cervical elasticity score at 12-14 weeks of pregnancy was greater than that predicted by the cervical length at the same time, and the area under the curve of cervical insufficiency predicted by the elasticity score and pre-pregnancy body mass index during the same period was the largest.

**Conclusions:** The cervical elasticity score at 12-14 weeks of gestation effectively predicted the occurrence of cervical insufficiency. The combination of the cervical elasticity score and pre-pregnancy body mass index predicted cervical insufficiency in women with twin pregnancies.

## Introduction

Cervical insufficiency (cervical incompetence, CI) is painless, progressive dilatation of the cervix before 37 gestational weeks, which can lead to premature rupture of the membranes, miscarriage, and preterm delivery 1. The prevalence of CI is 0.05-1% 2, but CI is associated with 14.3-65% of preterm births 3; therefore, assessing and predicting CI should be a concern. Transvaginal ultrasonography (TVU) is widely used to assess cervical function due to its diagnostic value in singleton pregnancies with CI 4-7. The evaluation includes measurements of cervical length and cervical elastography. Cervical length is predictive of CI and preterm delivery in singleton pregnancies 8, 9; moreover, ultrasound elastography improves the prediction of CI 10, 11.

Twin pregnancies have a higher probability of CI than do singleton pregnancies 12. However, only a few studies have focused on cervical assessments in twin pregnancies. Nevertheless, some studies that used ultrasonography for predicting CI in twin pregnancies reported inconsistent results. For singleton pregnancies, the Society for Maternal Fetal Medicine (SMFM) guidelines recommend a cervical assessment between 16-24 weeks 5. However, there is currently insufficient evidence to suggest that multiple pregnancies can benefit from cervical assessment and there is no uniform strategy for screening. More evidence-based research is needed to reach consensus. Thus, our study analyzed the clinical data of women with twin pregnancies and CI. We explored the predictive value of TVU for CI and provide a reference for clinically assessing and predicting CI.

## Materials and Methods

Data were collected from 179 women with twin pregnancies with CI from January 1, 2020 to January 31, 2022 at Xiangya Hospital Central South University. Of these cases, 37 were excluded due to reproductive tract infections, serious comorbidities, complications, or incomplete data. This study finally included 142 cases and 142 randomly matched cases without CI. Data on the perioperative surgical conditions and outcomes were collected. All procedures performed in studies involving human participants were in accordance with the ethical standards of the institution, and written consent was obtained. The study was conducted in accordance with the Declaration of Helsinki, and the protocol was approved by the Medical Ethics Committee of the Xiangya Hospital Central South University (202212286).

The inclusion criteria were women with twin pregnancies, complete ultrasound imaging data from our hospital, and no combined serious internal or surgical diseases. The exclusion criteria were severe comorbidities or complications, including severe preeclampsia, placental abruption, twin-to-twin transfusion syndrome, severe internal or surgical diseases, reproductive tract or intrauterine infection, severe fetal malformations, or incomplete data.

CI was diagnosed using the American College of Obstetricians and Gynecologists criteria 4, including medical history, physical examination, and TVU, when the cervical length was < 2.5 cm.

The standardized measurement of cervical length was consistent with previous studies 5. The long axis of the cervix was shown, and the standard sagittal section of the cervix was clearly displayed. The shortest value was taken as the final result after three consecutive measurements.

**Figure 1:**
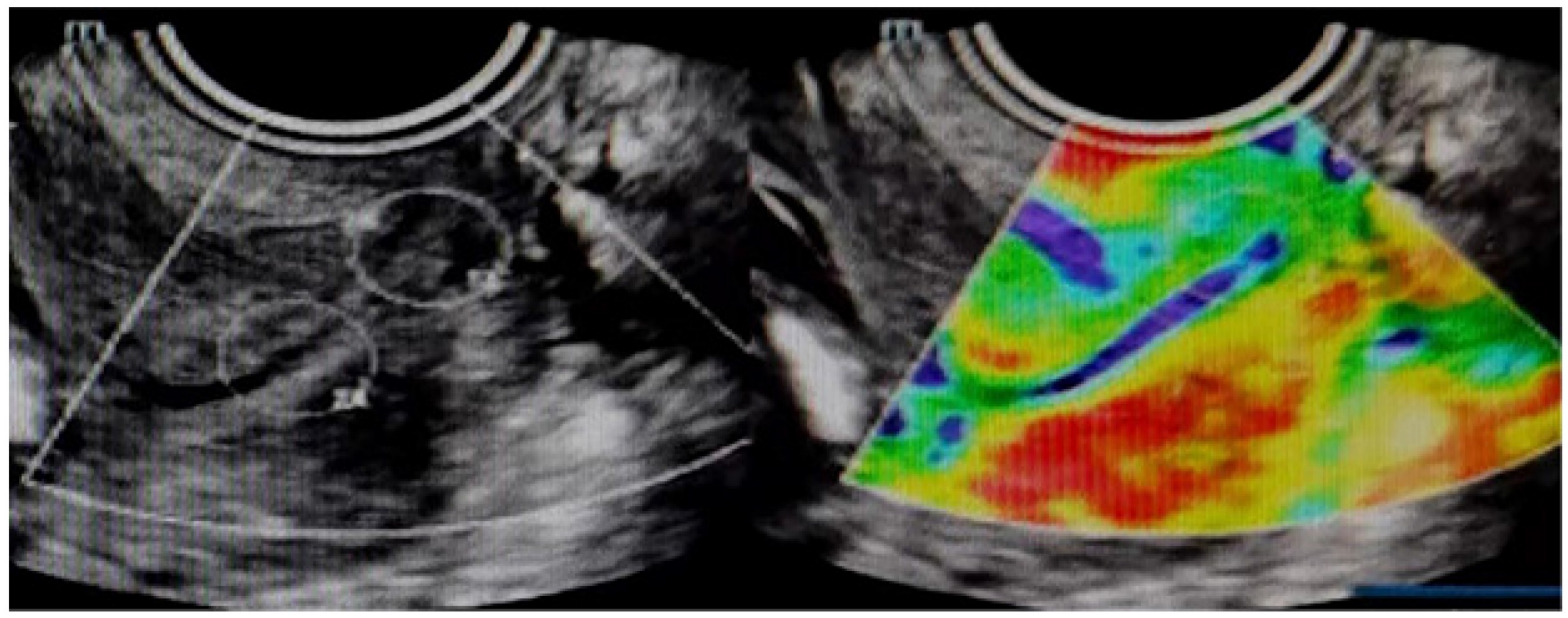
Elastography image with a score of 1.

The cervical elastography procedure was consistent with that of previous studies^13^. The basic ultrasound examination described above was performed. The elastogram is illustrated in green, red, and blue, with high hardness in blue, medium hardness in green, and low hardness in red. A score of 5 points was assigned when the target and surrounding area were blue,4 points when the overall performance of the target was blue, 3 points when the proportion of blue and green on the target was similar, 2 points when the surrounding area of the target was green and the center blue, and 1 point when the target was mostly green (Figure 1).The strain values at the endocervix, midpoint, and ectocervix were measured separately, and the average strain value was obtained by taking three consecutive measurements. The strain values of the ectocervix were used as a control to calculate the ratio of the inner/outer and middle/outer cervix.

Statistical analysis was performed using SPSS 26 software. The measurements are expressed as the mean±SD, and the counts are expressed as n (%). The statistical methods are t test and χ2-test. A P-value < 0.05 was considered significant. SPSS software was used to plot the receiver operating characteristic (ROC) curve and calculate the area under the curve (AUC). Logistic regression was used to analyze the risk factors.

## Results

The risk factors for CI in twin pregnancies included age, pregnancy pattern (e.g., assisted reproduction), cervical mechanical dilatation or injury, and history of polycystic ovarian syndrome (PCOS). History of second-trimester miscarriage or induction, which is a diagnostic criterion for CI, was significantly different between the CI and no-CI groups (Table 1). Follow-up logistic regression analysis showed that among the different risk factors, age was not a significant risk factor for the occurrence of CI (Table 2).

**Figure 2:**
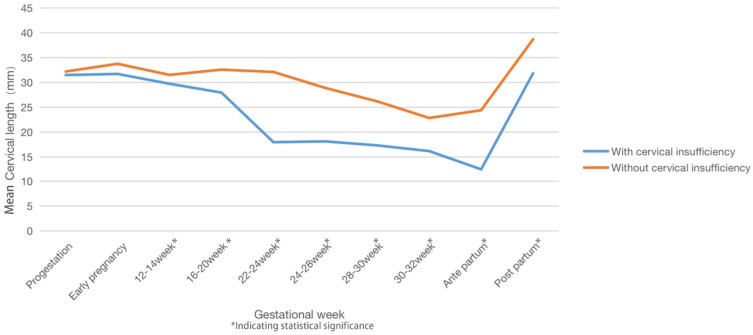
Changes in cervical length during a twin pregnancy.

**Figure 3:**
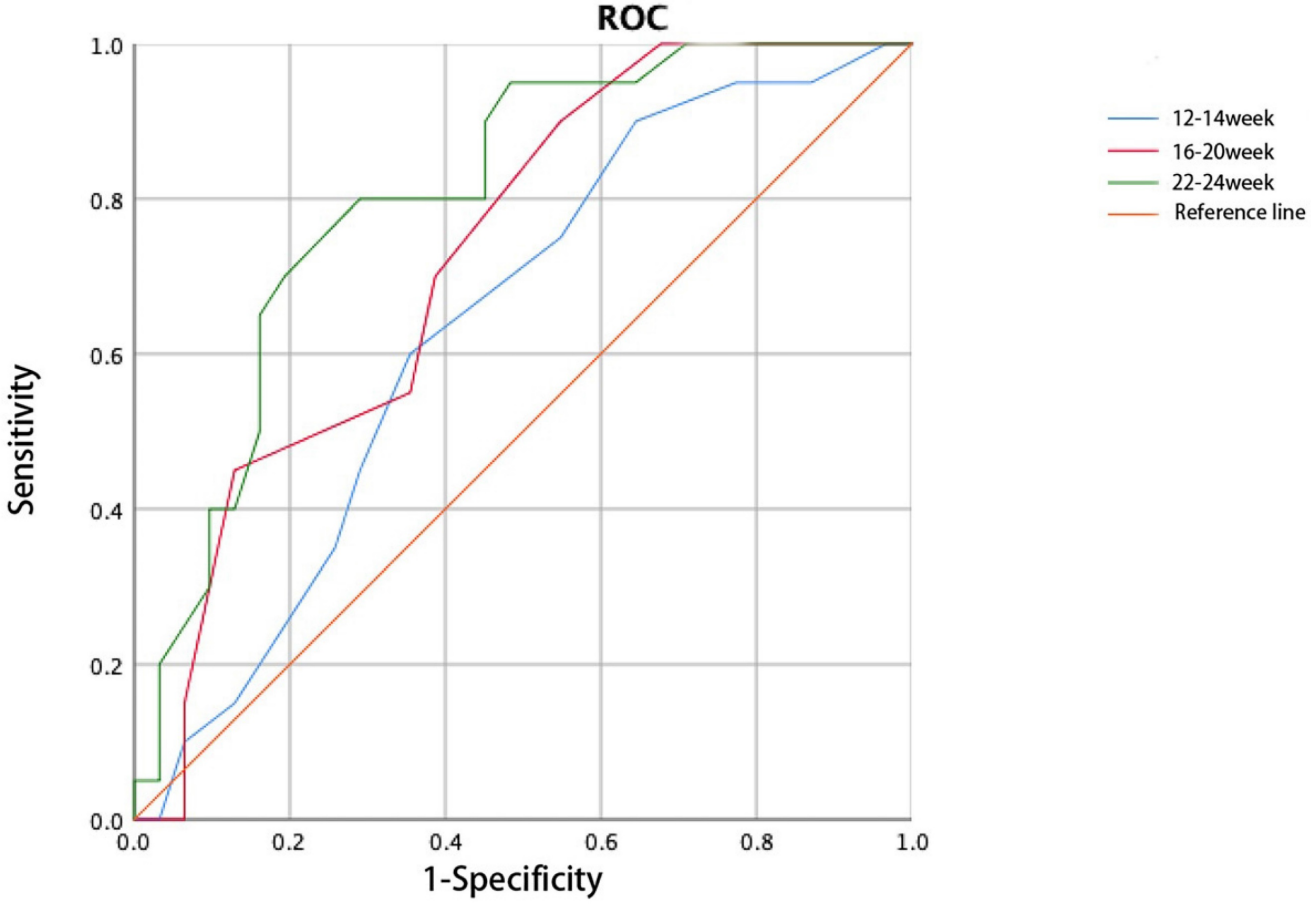
Receiver operating characteristic curve for cervical length predicts cervical insufficiency.

Cervical length decreased during pregnancy in both groups, and a significant difference in cervical length was detected between the two groups from 12-14 gestational weeks to postpartum (Table 3). The cervix was significantly shorter at 22-24 gestational weeks in women with twin pregnancies and CI than in those without CI. The cervical length recovered postpartum in both groups (Figure 2). Thus, cervical length between 12 and 24 weeks of gestation was used to construct the ROC curve, and the cervical length at 22-24 weeks was used to predict CI with the largest AUC (0.805, Figure 3).

**Figure 4:**
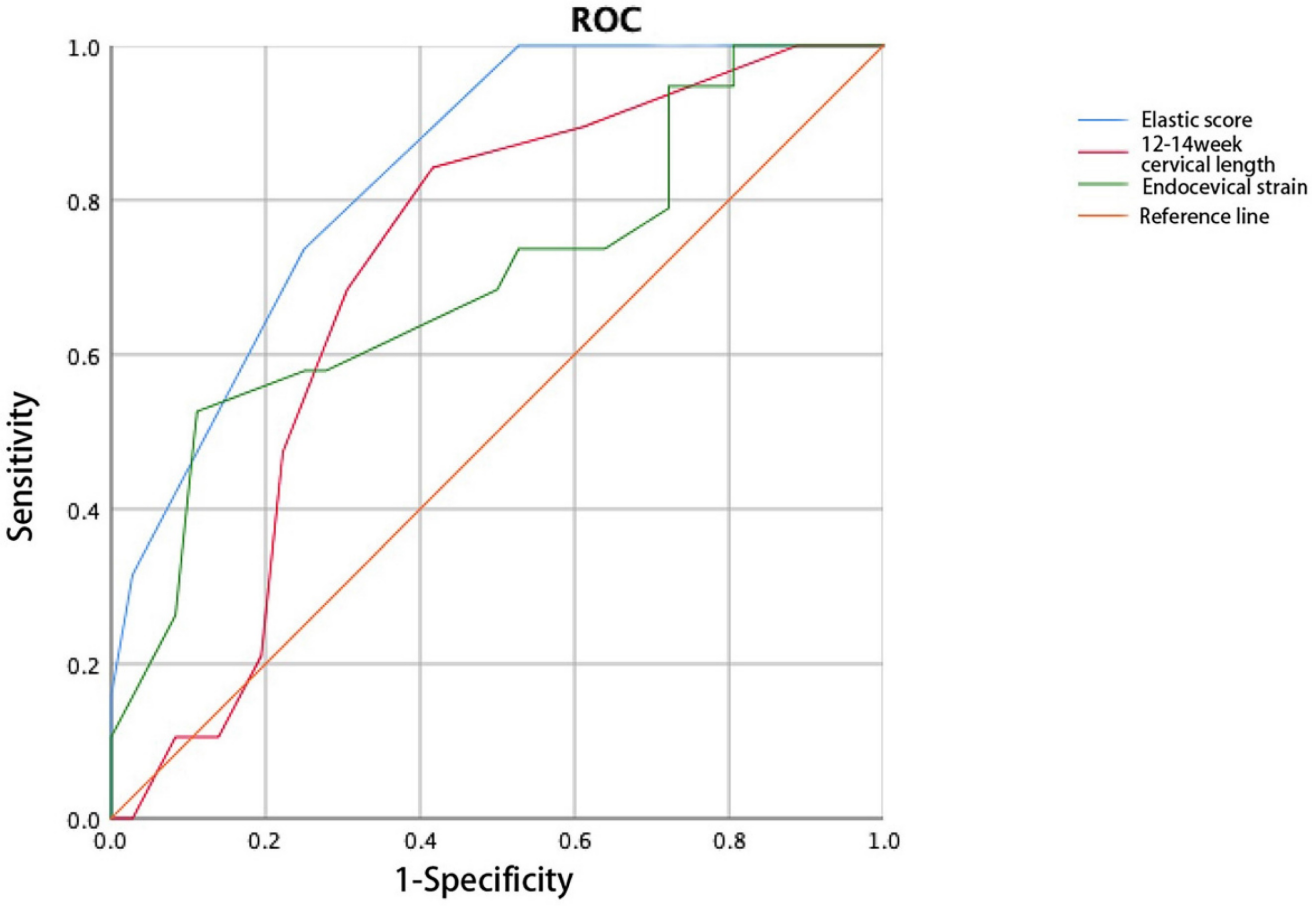
Receiver operating characteristic curve of the elasticity score predicts cervical insufficiency.

Cervical elastography was performed at 12-14 weeks of gestation. Significant differences in the cervical elasticity score and cervical internal orifice strain were detected between the two groups (Table 4). Thus, the 12-14-week elasticity score can be used to predict the occurrence of CI during twin pregnancies, and the AUC (0.837) was greater than that of cervical length during the same period (Figure 4).

**Figure 5:**
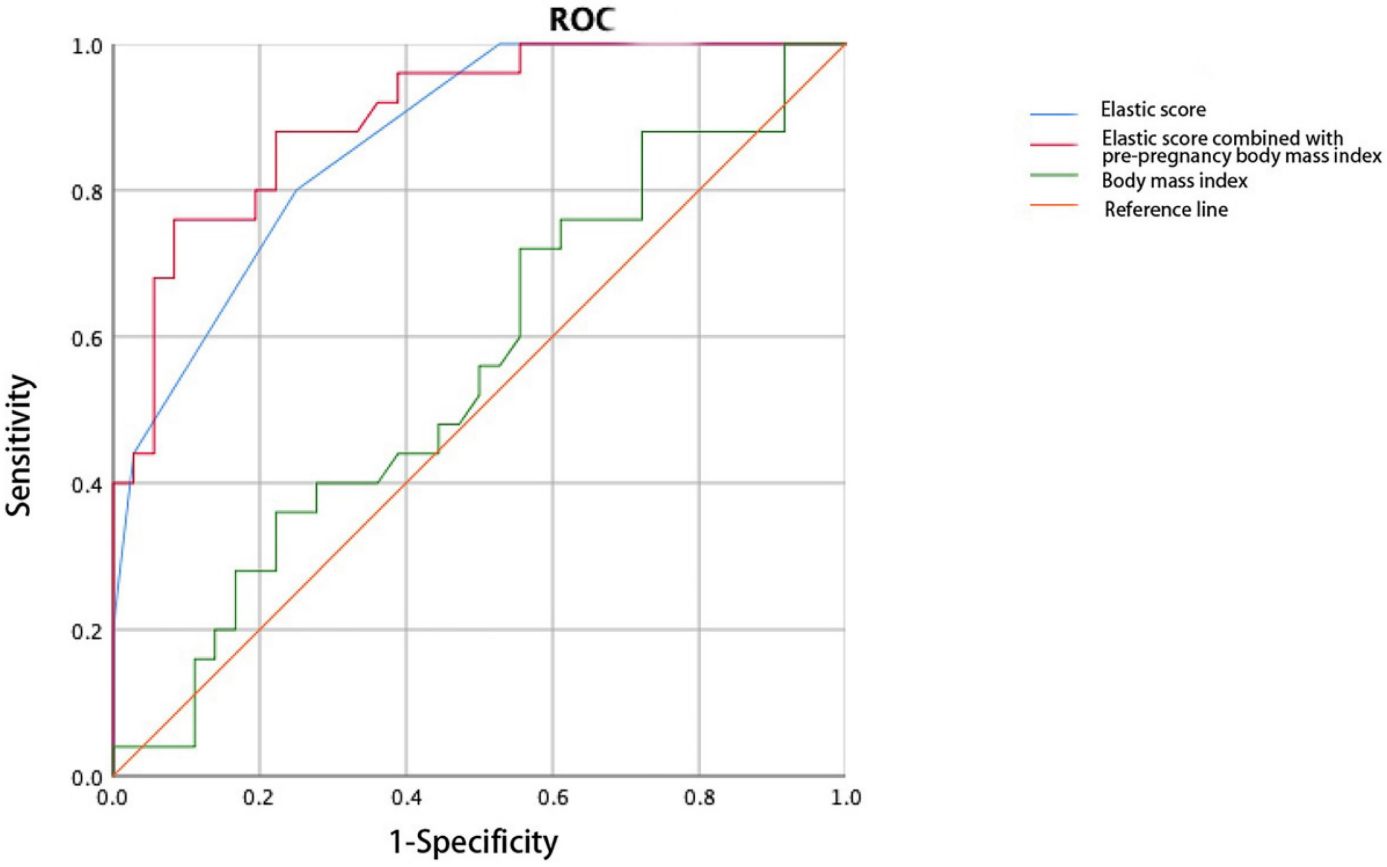
Receiver operating characteristic curve of the elasticity score combined with pre-pregnancy body mass index predicts cervical insufficiency.

**Figure 6:**
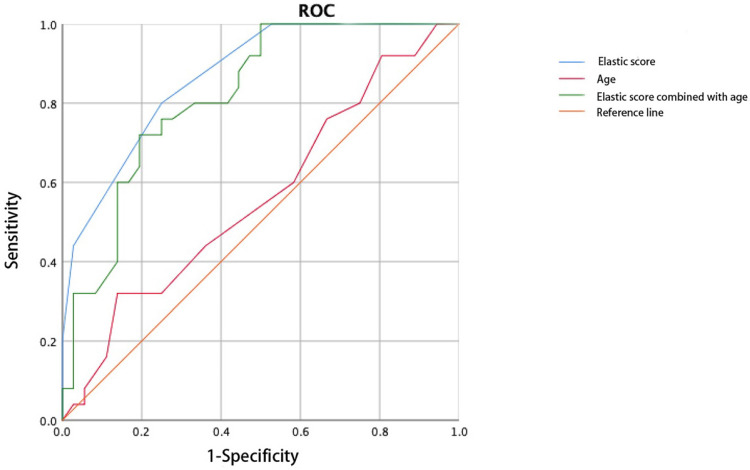
Receiver operating characteristic curve of the elasticity score combined with age predicts cervical insufficiency.

The cervical elasticity score was combined with age and pre-pregnancy body mass index (BMI) to construct the ROC curve. The AUC of the elasticity score combined with the pre-pregnancy BMI (0.902) to predict CI was larger than that of the elasticity score alone. The combination of the elasticity score and age did not improve the accuracy of predicting CI (AUC: 0.818, Figure 5, Figure 6).

## Discussion

This study showed that the risk factors for CI in women with twin pregnancies included assisted reproduction, cervical cold knife conization or loop electrosurgical excision procedure, a history of a uterine cavity operation, PCOS, and age. The above factors are consistent with those reported for singleton pregnancies 14-16.

There is insufficient evidence that cervical length during the first trimester can be used to predict CI and preterm birth. Similarly, the results of this study suggest that cervical length during the first trimester has limited predictive value. This study showed a difference in cervical length between the two groups at 16-20 weeks, but the ability of cervical length to predict CI was weak, which is consistent with the findings of Ashley et al. regarding singleton pregnancies 17. The difference in cervical length between the two groups may have been because some patients were diagnosed with CI due to a shortened cervix before 16-20 weeks, which requires further study.

Some results suggest that the cut-off value of cervical length in twin pregnancies is inconsistent 18, 19, so the same standards are not suitable for twin pregnancies and singleton pregnancies. Cervical length changes less before 20-24 weeks of pregnancy 6, 20. As gestation continues, the shortening of the cervix becomes non-linear. A study on twin pregnancies found that the factors that affect premature birth include the shortening rate, time that shortening started, and initial cervical length 21. These studies showed that cervical length changes continuously. We evaluated cervical length during twin pregnancies and showed that it shortened at 22-24 weeks, which is consistent with studies of singleton pregnancies. We found that the cervical length of women with twin pregnancies and CI stabilized after shortening at 22-24 weeks. Cervical length re-shortened before birth, but the range was less than that at 22-24 weeks. This may be because most patients were diagnosed with cervical insufficiency at 22-24 weeks. Therefore, our research focus on early prediction and aims to assess the risk of future CI pregnant women before 22-24 weeks. Continuously measuring cervical length can upgrade the evaluation of cervical function from static to dynamic, rather than obtaining a single value, which may be useful to evaluate women with a high CI risk more accurately.

The occurrence of CI is a premature cervical process. The changes in collagen composition during reshaping initially soften the cervix, and then the cervix expands and shortens due to reduced tolerance of the softened cervix 22. Therefore, women with a CI risk can be identified earlier by softening of the cervix than by the length measurement. Pre-pregnancy cervical elastography is useful to identify pregnant women with a CI risk 23, 24, suggesting that cervical elastography has a potential role in CI prediction. The Society for Maternal Fetal Medicine (SMFM) suggested that the timing of cervical length examination is between 16-24 weeks 5. Based on the theoretical basis that changes in cervical elasticity occur before cervical length, this study sets the time for cervical elastography examination to be 12-14 weeks.

We used strain elastography in this study. Previous studies have shown that various parameters of cervical strain elastography such as elastic contrast index, cervical external strain, cervical internal strain, have value for predicting premature birth from CI in singleton pregnancies, and their efficacy is better than that of cervical length 10, 11. This is partially consistent with our research, which found that only strain value at the endocervix is meaningful for predicting CI in twin pregnancies. We further analyzed the Strain value at the endocervix in predicting CI in the ROC curve of Figure 4. Its area is smaller than the cervical elasticity score, so we think that the Strain value at the endocervix monitored at 12-14 weeks of pregnancy has a weaker ability to predict future CI occurrence. Therefore, cervical elastography at 12-14 weeks helps identify women with high CI risk during twin pregnancies, but it cannot be used as the sole predictive factor. A study on singleton pregnancies showed that the elasticity score can be used to effectively predict premature birth caused by CI 25. This is similar to the results of our study on twin pregnancies, and its predictive effect was better than that of cervical length during the same period.

Age combined with the cervical elasticity score did not improve the efficacy of predicting CI in twin pregnancies, and the predictive value of cervical length did not change when combined with weight, age, or race 26, 27. The combination of the BMI and cervical elasticity score in twin pregnancies was superior to that of the elasticity score alone in predicting CI, similar to studies on singleton pregnancies 28, 29. We speculate that medical history factors have a greater effect on CI in singleton pregnancies, while a twin pregnancy itself is a risk factor for CI, and the effect of age may be weaker in this case; this needs to be confirmed.

Regarding the management of CI, the first step is to identify high-risk individuals through predictive methods and monitor cervical length more frequently during pregnancy, hoping to detect painless cervical shortening or dilation as early as possible. Patients who meet the indications should undergo preventive cervical cerclage. If cervical dysfunction occurs, measures should be taken as early as possible before abortion, such as cervical cerclage, progesterone.

One of the limitations of this study is the subjectivity of elastography. However, subsequent training may reduce this error. In Table 1, some factors such as age showed statistically significant differences between the two groups. Although previous studies have shown that age is one of the risk factors for CI 30, further research is needed to confirm whether this conclusion applies to twin pregnancies. Due to the different units and measurement methods for various items, different statistical methods were used in Table 1. For quantitative items, we used mean and standard deviation to represent them, while for qualitative items, we used prevalence rates to represent them. The results do not affect the statistical significance of the item.

As a single-center retrospective study, the number of patients was limited, and future prospective multi-center studies are expected.

In this study, we discuss the risk factors, cervical measurements, and ultrasound elastography parameters for predicting CI during twin pregnancies. Overall, assisted reproduction, cervical cold knife conization or loop electrosurgical excision procedure, history of uterine cavity operation, and PCOS were risk factors for CI in twin pregnancies. The combination of the cervical elasticity score at 12-14 weeks of gestation with pre-pregnancy BMI was useful for predicting CI in twin pregnancies.

## Funding

This research was funded by the Natural Science Foundation of Hunan Province (2022JJ40789, 2023JJ40980, 2023JJ40958), the China Postdoctoral Science Foundation (2022M723555), the National Natural Science Foundation of China (82301927, 82371700, 81974236, 81571516, 81903696), the Major Scientific and Technological Projects for Collaborative Prevention and Control of Birth Defects in Hunan Province (2019SK1010, 2019SK1015), the Key Research and Development Program of Hunan Province (2020SK2072).

## Figures and Tables

**Table 1:** Comparison of basicdatain twin pregnancies with or without CI

Variable	Twin pregnancies with CI	Twin pregnancies without CI	P
Age, years (mean±SD)	31.54±4.34	30.5±3.96	0.037
Body mass index (BMI), kg/m^2^ (mean±SD)	22.51±3.21	21.95±2.57	0.105
Assisted reproductive technology (ART), n (%)	131 (92.25%)	109 (76.76%)	<0.001
Types of chorion			0.105
Dichorionic diamniotic, n (%)	129 (90.85%)	120 (84.51%)	
Monochorionic diamniotic, n (%)	13 (9.15%)	22 (15.49%)	
Uterine cavity operation (mean±SD)	1.65±1.80	0.80±1.11	<0.001
Hysteroscopy (mean±SD)	1.20±1.57	0.37±0.81	<0.001
Uterine curettage (mean±SD)	0.46±0.84	0.43±0.78	0.717
Cold knife conization (CKC)/ loop electrosurgical excision procedure (LEEP), n (%)	19 (13.38%)	4 (2.82%)	0.002
Times of natural production (mean±SD)	0.23±0.51	0.16±0.42	0.256
History of second-trimester miscarriage or induction, n (%)	28 (19.72%)	10 (7.04%)	0.006
Polycystic ovarian syndrome (PCOS), n (%)	25 (17.61%)	9 (6.34%)	0.003
Gestational diabetes mellitus (GDM), n (%)	20 (14.08%)	32 (22.54%)	0.066

**Table 2:** Multivariate logistic regression analysis of risk factors

	P	OR	95%Confidence interval
Assisted reproductive technology (ART)	0.02	3.161	1.199-8.334
Hysteroscopy	0.002	3.831	1.666-8.811
Cold knife conization (CKC)/ loop electrosurgical excision procedure (LEEP)	0.012	4.388	1.389-13.864
History of second-trimester miscarriage or induction	0.050	2.312	0.993-5.386
Polycystic ovarian syndrome (PCOS)	0.004	3.759	1.533-9.220
Age	0.306	1.039	0.966-1.118
Body mass index (BMI)	0.164	1.070	0.973-1.176

**Table 3:** Cervical length at different periods in twin pregnancies

Cervical length (mean±SD)	Twin pregnancies with CI	Twin pregnancies without CI	P
Progestation	31.45±2.84	32.35±3.85	0.052
Early pregnancy	31.72±3.92	33.77±4.67	0.070
12-14 week	29.74±3.22	31.50±2.58	0.01
16-20 week	27.95±7.80	32.40±2.65	<0.001
22-24 week	17.94±10.16	31.92±4.55	<0.001
24-28 week	18.10±9.32	28.90±4.69	<0.001
28-30 week	17.29±8.48	26.18±7.83	<0.001
30-32 week	16.14±7.05	22.83±8.35	0.001
Antepartum	12.45±6.97	24.4±5.59	<0.001
Postpartum	31.86±6.64	38.38±5.41	<0.001

**Table 4:** Elasticity parameters in twin pregnancies with versus without cervical insufficiency

	Twin pregnancies with CI	Twin pregnancies without CI	P
Elasticity score	2.39±0.71	2.97±0.65	0.010
Strain value at the endocervix	0.14±0.08	0.09±0.04	0.032
Strain value at the midpoint	0.24±0.10	0.19±0.09	0.105
Strain value at the ectocervix	0.29±0.10	0.26±0.13	0.322
The strain value ratio of the inner/outer cervix	2.54±0.92	3.04±1.05	0.117
The strain value ratio of the middle/outer cervix	1.33±0.47	1.61±0.88	0.168

## References

[1] Brown R, Gagnon R, Delisle MF (2019). No. 373-Cervical Insufficiency and Cervical Cerclage. J Obstet Gynaecol Can.

[2] Wang Y, Gu X, Tao L, Zhao Y (2016). Co-morbidity of cervical incompetence with polycystic ovarian syndrome (PCOS) negatively impacts prognosis: A retrospective analysis of 178 patients. BMC Pregnancy Childbirth.

[3] Barinov SV, Artymuk NV, Novikova ON, Shamina IV, Tirskaya YI, Belinina AA (2021). Analysis of risk factors and predictors of pregnancy loss and strategies for the management of cervical insufficiency in pregnant women at a high risk of preterm birth. J Matern Fetal Neonatal Med.

[4] ACOG Practice Bulletin No.142 (2014). Cerclage for the management of cervical insufficiency. Obstet Gynecol.

[5] McIntosh J, Feltovich H, Berghella V, Manuck T (2016). The role of routine cervical length screening in selected high- and low-risk women for preterm birth prevention. Am J Obstet Gynecol.

[6] Gudicha DW, Romero R, Kabiri D, Hernandez-Andrade E, Pacora P, Erez O (2021). Personalized assessment of cervical length improves prediction of spontaneous preterm birth: a standard and a percentile calculator. Am J Obstet Gynecol.

[7] Zhang MY, Zhang XX, Yang HX, Shi CY (2021). Cervical length at 28-32 weeks of gestation predicts preterm birth. Matern Fetal Med.

[8] Leung TN, Pang MW, Leung TY, Poon CF, Wong SM, Lau TK (2005). Cervical length at 18-22 weeks of gestation for prediction of spontaneous preterm delivery in Hong Kong Chinese women. Ultrasound Obstet Gynecol.

[9] El-Ardat MA, Gavrankapetanovic F, Abou El-Ardat KA, Dekovic S, Murtezic S, Mehmedbasic E (2014). Ultrasound measurement of cervical length as predictor of threatened preterm birth: a predictive model. Acta Inform Med.

[10] Park HS, Kwon H, Kwak DW, Kim MY, Seol HJ, Hong JS, Shim JY (2019). Addition of Cervical Elastography May Increase Preterm Delivery Prediction Performance in Pregnant Women with Short Cervix: a Prospective Study. J Korean Med Sci.

[11] Du L, Zhang LH, Zheng Q, Xie HN, Gu YJ, Lin MF (2020). Evaluation of Cervical Elastography for Prediction of Spontaneous Preterm Birth in Low-Risk Women: A Prospective Study. J Ultrasound Med.

[12] Payne MS, Newnham JP, Doherty DA, Furfaro LL, Pendal NL, Loh DE (2021). A specific bacterial DNA signature in the vagina of Australian women in midpregnancy predicts high risk of spontaneous preterm birth (the Predict 1000 study). Am J Obstet Gynecol.

[13] Zhi H, Ou B, Xiao XY, Peng YL, Wang Y, Liu LS (2013). Ultrasound elastography of breast lesions in chinese women: a multicenter study in China. Clin Breast Cancer.

[14] Feigenbaum SL, Crites Y, Hararah MK, Yamamoto MP, Yang J, Lo JC (2012). Prevalence of cervical insufficiency in polycystic ovarian syndrome. Hum Reprod.

[15] Kyrgiou M, Athanasiou A, Paraskevaidi M, Mitra A, Kalliala I, Martin-Hirsch P (2016). Adverse obstetric outcomes after local treatment for cervical preinvasive and early invasive disease according to cone depth: systematic review and meta-analysis. BMJ.

[16] Saccone G, Zullo F, Roman A, Ward A, Maruotti G, Martinelli P (2019). Risk of spontaneous preterm birth in IVF-conceived twin pregnancies. J Matern Fetal Neonatal Med.

[17] Hester AE, Ankumah NE, Chauhan SP, Blackwell SC, Sibai BM (2019). Twin transvaginal cervical length at 16-20 weeks and prediction of preterm birth. J Matern Fetal Neonatal Med.

[18] Prodan N, Wagner P, Sonek J, Abele H, Hoopmann M, Kagan KO (2020). Single and repeat cervical-length measurement in twin gestation with threatened preterm labor. Ultrasound Obstet Gynecol.

[19] Roman A, Ramirez A, Fox NS (2022). Screening for preterm birth in twin pregnancies. Am J Obstet Gynecol MFM.

[20] Souka AP, Papastefanou I, Michalitsi V, Salambasis K, Chrelias C, Salamalekis G (2011). Cervical length changes from the first to second trimester of pregnancy, and prediction of preterm birth by first-trimester sonographic cervical measurement. J Ultrasound Med.

[21] Melamed N, Pittini A, Hiersch L, Yogev Y, Korzeniewski SS, Romero R (2016). Serial cervical length determination in twin pregnancies reveals 4 distinct patterns with prognostic significance for preterm birth. Am J Obstet Gynecol.

[22] Vink J, Myers K (2018). Cervical alterations in pregnancy. Best Pract Res Clin Obstet Gynaecol.

[23] Öcal FD, Çekmez Y, Erdoğdu E, Gezer M, Fanuscu İ, Özkan H (2015). The utility of cervical elastosonography in prediction of cervical insufficiency: cervical elastosonography and cervical insufficiency. J Matern Fetal Neonatal Med.

[24] Zhang L, Zheng Q, Xie H, Du L, Wu L, Lin M (2020). Quantitative cervical elastography: a new approach of cervical insufficiency prediction. Arch Gynecol Obstet.

[25] Gesthuysen A, Hammer K, Möllers M, Braun J, Oelmeier de Murcia K, Falkenberg MK (2020). Evaluation of Cervical Elastography Strain Pattern to Predict Preterm Birth. Ultraschall Med.

[26] Klein K, Gregor H, Hirtenlehner-Ferber K, Stammler-Safar M, Witt A, Hanslik A (2008). Prediction of spontaneous preterm delivery in twin pregnancies by cervical length at mid-gestation. Twin Res Hum Genet.

[27] Mancuso MS, Szychowski JM, Owen J, Hankins G, Iams JD, Sheffield JS (2010). Cervical funneling: effect on gestational length and ultrasound-indicated cerclage in high-risk women. Am J Obstet Gynecol.

[28] Wilms FF, Vis JY, de Wit-Zuurendonk L, Porath MM, Mol BW (2015). What is the risk of preterm delivery after arrested preterm labor. Am J Perinatol.

[29] Kindinger LM, Poon LC, Cacciatore S, MacIntyre DA, Fox NS, Schuit E (2016). The effect of gestational age and cervical length measurements in the prediction of spontaneous preterm birth in twin pregnancies: an individual patient level meta-analysis. BJOG.

[30] Meng L, Öberg S, Sandström A, Wang C, Reilly M (2022). Identification of risk factors for incident cervical insufficiency in nulliparous and parous women: a population-based case-control study. BMC Med.

